# Supervised Relation Extraction Between Suicide-Related Entities and Drugs: Development and Usability Study of an Annotated PubMed Corpus

**DOI:** 10.2196/41100

**Published:** 2023-03-08

**Authors:** Karina Karapetian, Soo Min Jeon, Jin-Won Kwon, Young-Kyoon Suh

**Affiliations:** 1 School of Computer Science and Engineering Kyungpook National University Daegu Republic of Korea; 2 College of Pharmacy Jeju National University Jeju Republic of Korea; 3 BK21 FOUR Community-Based Intelligent Novel Drug Discovery Education Unit, College of Pharmacy and Research Institute of Pharmaceutical Sciences Kyungpook National University Daegu Republic of Korea

**Keywords:** suicide, adverse drug events, information extraction, relation classification, bidirectional encoder representations from transformers, pharmacovigilance, natural language processing, PubMed, corpus, language model

## Abstract

**Background:**

Drug-induced suicide has been debated as a crucial issue in both clinical and public health research. Published research articles contain valuable data on the drugs associated with suicidal adverse events. An automated process that extracts such information and rapidly detects drugs related to suicide risk is essential but has not been well established. Moreover, few data sets are available for training and validating classification models on drug-induced suicide.

**Objective:**

This study aimed to build a corpus of drug-suicide relations containing annotated entities for drugs, suicidal adverse events, and their relations. To confirm the effectiveness of the drug-suicide relation corpus, we evaluated the performance of a relation classification model using the corpus in conjunction with various embeddings.

**Methods:**

We collected the abstracts and titles of research articles associated with drugs and suicide from PubMed and manually annotated them along with their relations at the sentence level (adverse drug events, treatment, suicide means, or miscellaneous). To reduce the manual annotation effort, we preliminarily selected sentences with a pretrained zero-shot classifier or sentences containing only drug and suicide keywords. We trained a relation classification model using various Bidirectional Encoder Representations from Transformer embeddings with the proposed corpus. We then compared the performances of the model with different Bidirectional Encoder Representations from Transformer–based embeddings and selected the most suitable embedding for our corpus.

**Results:**

Our corpus comprised 11,894 sentences extracted from the titles and abstracts of the PubMed research articles. Each sentence was annotated with drug and suicide entities and the relationship between these 2 entities (adverse drug events, treatment, means, and miscellaneous). All of the tested relation classification models that were fine-tuned on the corpus accurately detected sentences of suicidal adverse events regardless of their pretrained type and data set properties.

**Conclusions:**

To our knowledge, this is the first and most extensive corpus of drug-suicide relations.

## Introduction

### Background

Suicide is an intentional death that is caused by self-harm. Although global suicide rates have declined in recent years, suicide accounts for approximately 700,000 deaths (1.3% of all deaths) per annum [1]. The Comprehensive Mental Health Action Plan (2013-2020) of the World Health Organization argues that suicide remains a critical global public health problem [1].

Although suicide can be triggered by multiple factors and their complex effects [2], most cases are related to psychiatric disorders such as depression, psychosis, anxiety, and substance use [3]. Physical disorders such as cancer, respiratory diseases, hypertension, and diabetes are also debated as risk factors for suicide [4,5]. Effective treatment of individual patients can avoid and decrease the suicide risk associated with these factors; however, caution is required because the prescribed drug may itself be an independent risk factor for suicide.

Several studies have suggested a link between suicidal behaviors (suicidal ideation, attempted suicide, and completed suicide) and adverse events associated with prescribed drugs [6-9]. For instance, a previous meta-analysis of clinical trials showed that selective serotonin reuptake inhibitors (SSRIs) tend to increase the risk of suicidality in patients with depression and all indications [10]. Consequently, the United States Food and Drug Administration issued a black box warning for the suicidal risk of SSRIs. Qato et al [11] investigated the use of drugs that pose a potential suicide risk in the United States. They reported 103 drugs associated with suicidality as an adverse event; furthermore, the use of these drugs substantially increased from 17.3% in 2005-2006 to 23.5% in 2013-2014 [11].

To prevent and reduce the occurrence of drug-induced suicide, we must improve our knowledge of the drugs that pose a potential suicide risk. Although clinical trials have evaluated the efficacy and safety of drugs in the premarketing phase, they usually have strict inclusion and exclusion criteria, short-term duration, and small sample size, which limit their ability to detect *rare* adverse drug events (ADEs) [12-14]. Therefore, ongoing evaluations of drugs introduced to the market, called postmarketing surveillance, are crucial for rare ADEs such as suicide.

### Theoretical Background

Among various sources of information on ADEs in the postmarketing surveillance field, research articles are the most informative. However, extracting such information from these data sources is challenging because it is recorded in an unstructured free-text format.

Automatic information extraction systems can be developed through natural language processing (NLP), a field of computer science and artificial intelligence. A system that automatically excerpts information from research articles can accelerate the task of identifying drugs with potential suicide risk.

The most general purpose corpora for relation extraction tasks in the biomedical domain contain diverse entities and relations [15-17]. More narrowly focused data sets represent the interactions between diseases [18], drugs [19], chemical components and diseases [20], and drug and ADEs [21,22]. However, these corpora contain insufficient data when developing an information extraction system for drug-related suicidal events. For instance, the MEDLINE ADE data set contains only 3 (0.04%) suicide-related entities among 6821 sentences. These sentences are presented in Multimedia Appendix 1 [15,21-23].

Several studies have attempted to classify sequences as suicide-related or nonsuicide-related sentences [24-26]. Such fixed relation agents require information on the agents themselves in the data set because the model must learn the entities between which the relation should be classified. Furthermore, models developed using the data sources of social media may not be adjustable to data from research articles, mainly because scientific texts follow strict grammatical rules rather than social language [27], which is characterized by a high rate of abbreviations, nonformal terminology, and metaphoric phrases [28].

### Related Work

As drug-induced suicide is a type of ADE, we reviewed the published data sets on ADEs. Most of these data sets contain information on drugs and conditions (eg, diseases, signs, and symptoms) and the relationship between these entities. Nikfarjam et al [23] created the ADRMine data set from posts on Twitter and the health-related social network DailyStrength [29]. They annotated signs and symptoms at the sentence level, including adverse drug reactions. Van Mulligen et al [15] created the EU-ADR corpus from the titles and abstracts of MEDLINE articles. They annotated the drugs and diseases and the relationship between these entities. For instance, a *drug-disease* relation in their corpus indicates that the drug may produce an adverse effect at the sentence level but does not necessarily imply an ADE. Schulz et al [16] developed another corpus based on case reports from PubMed. They annotated the cases, conditions, findings, factors, negation modifiers, and relationship between these entities. Gurulingappa et al [21] developed a MEDLINE ADE corpus to support the automatic extraction of drug-related adverse events from case reports in MEDLINE (a subset of PubMed). Their corpus contains 4272 unique sentences and 6821 relations. Alvaro et al [22] created a source-comparative corpus called TwiMed, which includes annotated drugs, symptoms, diseases, and negative drug-associated outcomes. Multimedia Appendix 2 provides a detailed comparison of these corpora.

### Problem

Several studies have produced various drug-related corpora and general diseases. However, as the existing corpora seldom focus on drug-induced suicide events, we cannot gain extensive knowledge of medicines that pose a potential risk of suicide. This knowledge gap limits our ability to prevent and reduce the occurrence of drug-related suicides. Moreover, few corpora include the directional relationship between drugs and suicide and vice versa. To address these concerns, we constructed a novel drug-suicide relation (DSR) corpus from a wide range of biomedical articles on PubMed.

### Objective

The objective of our research was to construct a DSR corpus. The obtained corpus consisted of 11,894 sentences extracted from PubMed research articles. It included (1) annotations on 2 entities (drug and suicidal events) and (2) annotations on the relations between the entities. PubMed provides access to broad-spectrum articles in the biomedical field, covering >70% of all publications [30]. Therefore, our corpus may be useful for developing information extraction models for diverse biomedical databases. To validate our corpus, we evaluated the relation classification performances of Bidirectional Encoder Representations from Transformer (BERT) models fine-tuned on data sets with diverse properties extracted from our corpus.

## Methods

### Overview

**Figure 1:**
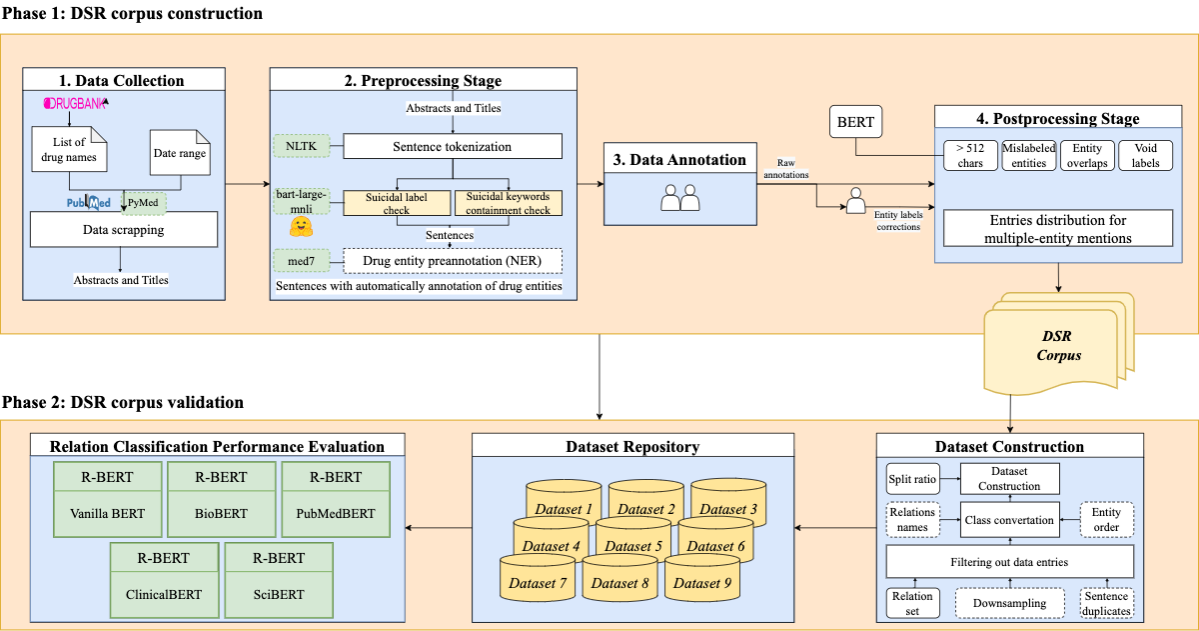
Workflow of constructing and testing the DSR (drug-suicide relation) corpus. BERT: Bidirectional; Encoder Representations from Transformer; NER: named entity recognition; NLTK: natural language toolkit.

This study was conducted in two phases: (1) construction of the DSR corpus and (2) validation of the DSR corpus. To implement the first phase, we developed a sophisticated workflow comprising four steps: (1) data collection, (2) preprocessing stage, (3) data annotation, and (4) postprocessing stage. First, we gathered data from DrugBank and PubMed and preprocessed them for further annotation. Second, we manually annotated the entity pairs and relation classes for each sentence. Third, we created the corpus from the raw annotations via postprocessing of the labeled data. We then built various data sets from the corpus with different parameters for the subsequent phase. In the second phase of our study, we evaluated the performance of the BERT-based relation classification model using several language models (LMs) fine-tuned on various data sets compiled from our developed corpus. Both phases were implemented using Python 3.7. Figure 1 shows the overall workflow for constructing and testing the DSR corpus.

### Generation of the DSR Corpus

#### Data Collection and Preprocessing

We collected the titles and abstracts of all available articles in English on the association between drugs and suicide published by October 13, 2021. Textbox 1 presents the search queries used in this study.

PubMed contains metadata at the level of a paper, which are useful for data filtering in the collection stage. When building the search query, we used the Medical Subject Headings (MeSH) terms [31] “suicidal ideation,” “suicide, attempted,” “suicide, completed,” and “suicide,” along with text words associated with the keyword “suicide” in PubMed. We considered generic drug names from DrugBank version 5.1.8 [32,33] and their synonyms for drugs. We excluded drugs categorized as vitamins, mineral supplements, tonics, blood substitutes, emollients and protectives, antiseptics and disinfectants, or medicated dressings according to the Anatomical Therapeutic Chemical Classification System [34] and various sections of the classification. We used *PyMed* package (version 0.8.9) [35] for PubMed to automate the task of collecting the titles and abstracts of articles associated with each drug.

The collected titles and abstracts were tokenized at the sentence level using a pretrained tokenizer in the *NLTK* package (version 3.6.1; [36]). Among the sentences obtained (N=172,249) from 17,017 articles on PubMed, we collected only those sentences containing information on drugs and suicide. The DSR corpus was then developed at the sentence level as follows: first, sentences containing at least one mention of a drug were selected. Second, we chose suicide-related sentences that (1) contained the suicidal keyword “suicid,” (a stemmed version of the word “suicide”) or (2) are classified as “suicidal” by a model. Yin et al [37] proposed a method using models pretrained on natural language understanding data sets as zero-shot sequence classifiers**.** To check whether the suicide-related sentences are classified as “suicidal,” we used a Bidirectional and Auto-Regressive Transformers (BART) large model [38] pretrained on the Multi-Genre Natural Language Inference corpus [39] with the custom binary classification of “suicide” and “non-suicide.” If the model infers that a given sentence is “suicide” with a probability of ≥.5, it assigns a suicidal label to that sentence. Finally, we obtained 9732 data entries for annotation.

**PubMed query template for retrieving drug-mentioning suicide-related articles.**

(%DRUG% [Supplementary concept] OR %DRUG%[MeSH Terms] OR %DRUG%[TW]) AND (“suicidal ideation”[MeSH Terms] OR “suicide, attempted”[MeSH Terms] OR “suicide, completed”[MeSH Terms] OR “suicide”[MeSH Terms] OR suicid[TW] OR suicidals[TW] OR suicidality[TW] OR suicide[TW] OR suicidal[TW] OR suiciders[TW] OR suicidally[TW] OR suicides[TW] OR suicide s[TW] OR suicided[TW]) AND

(English[Language])

#### Data Annotation

During the data annotation stage, our workflow assigned three labels to each sentence: (1) drug entity, (2) suicide entity, and (3) relation class. Two annotators with pharmacological backgrounds participated independently in the annotation process. First, 2 annotators reviewed the automatically annotated [40] labels of drug entities. The annotators assigned each drug’s generic name, brand name, class name, and abbreviated name as a drug entity. The metabolite and salt forms of the drug were excluded. Second, they manually annotated the suicide entities in each sentence. The suicidal entities were defined as mentions of suicide-related events, tendencies, and behaviors, including suicide risk, suicidal attempt, completed suicide, and suicidal ideation, or suicide-related behavior disorders. Third, they classified the relation class for each sentence as an “*adverse drug event* (ADE),” “*suicide means,*” “*treatment,*” “*miscellaneous*” (such as comparative sentences, research objectives, miscellaneous sentences, and no explicit relation), or “*none.*”

The primary relations between a drug and a suicidal entity were set as follows:

1. ADEs: This relation indicates that suicidal events, including suicide attempts, suicide completions, and self-harm–related behaviors, followed the drug administration.
2. Suicide means: This relation indicates that the drug was deliberately used (ie, taken in overdose) to commit suicide.
3. Treatment: This relation indicates that the drug was used to treat the signs or symptoms of suicidal ideation and suicidal behavior disorder.

**Figure 2:**
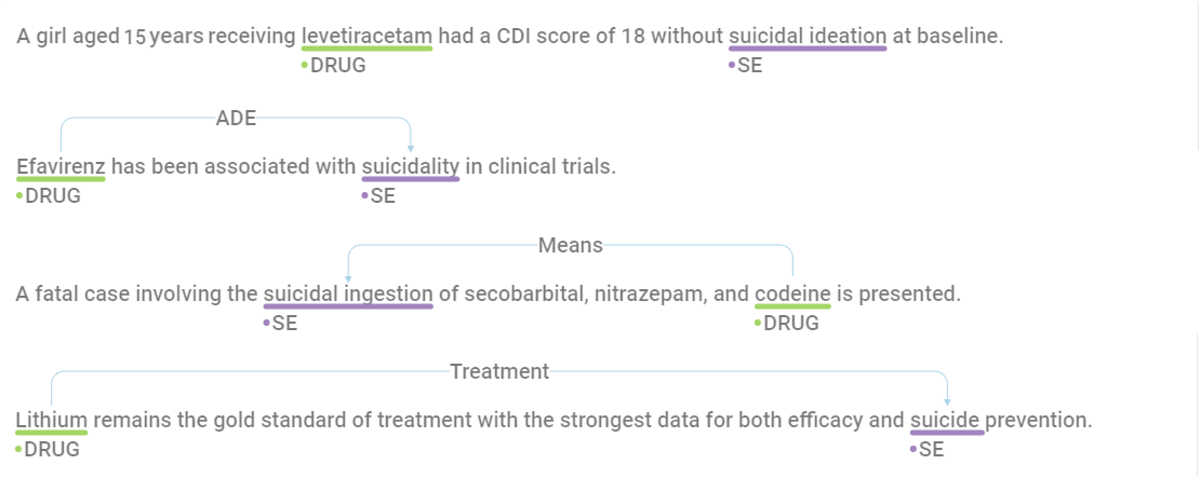
Examples of relation class entries: the sentences of each class in the Doccano environment are annotated. ADE: adverse drug event; CDI: Children's Depression Inventory; SE: suicidal entity.

When multiple entities for drugs or suicide appeared in a single sentence, we represented all “sentence–drug entity–suicidal entity” cases by duplicating the sentence. The “relation-class” label was excluded from the identifying representation because the relations between the same entities cannot overlap. The annotation guidelines are detailed in Multimedia Appendix 3 [40]. Figure 2 [41] shows some relation-class entries. Each data entry includes a sentence, drug entity, suicide-related entity, and the relation class between the 2 entities.

#### Interannotator Agreement

Two annotators with pharmacological backgrounds independently annotated the drug and suicide entities and their relations in each sentence. The annotators then compared their annotations and matched the annotations for drug and suicide entities according to the annotation guidelines (Multimedia Appendix 3). When a disagreement was observed, the annotations were matched by 2 independent reviewers (one pharmacist and the other with an NLP background). To validate the annotated relations between entities, we measured interannotator agreement using the Cohen κ method [42]. We aligned the proposed relation classes of the 2 annotators between the same pair of entities in the same sentence. The interannotator agreement score was calculated as a pairwise Cohen κ score. The data were annotated with a Cohen κ score of 0.64, implying a substantial level of agreement [43,44].

#### Annotation Postprocessing Process

In the postprocessing stage, we revised the annotations of labels completed by the annotators and adjusted the data format to be used for relation classification with the BERT models. Before this step, the data were sorted in ascending order of occurrence number of each sentence in the data set. This sorting procedure reduced the probability of choosing duplicates when constructing the data set (selecting specific classes and implementing the downsampling process). Meanwhile, we eliminated examples with (1) ambiguous annotations not related to suicide (such as “suicide gene” and “suicidal patients”) for the suicidal entity, (2) at least one missing value of assigned labels, (3) sentence lengths >512 characters (the maximum allowed by the vanilla BERT model) [45], (4) no mentions of the annotated entities in the sentence, or (5) overlapped entities. Excluding the examples with sentence lengths >512 characters was deemed acceptable as most (but not all) of the recent relation classification models [46-52] use BERT-based or RoBERTa-based approaches [53]. Although the BERT architecture of RoBERTa [53] has been optimized for faster learning, the maximum sequence length remains at 512 characters. Furthermore, such long sentences were few in our corpus; therefore, their impact was almost negligible. We then distributed the data records with multiple appearances of the same entity in a sentence and calculated the exact positions of the entities in the sentence. Finally, we obtained the final corpus with a size of 11,894.

### Validation of the DSR Corpus: Fine-tuning R-BERT Models for Relation Classification

#### Data Set Construction

For the relation classification experiments, we constructed several data sets based on our DSR corpus, removing duplicated sentences to avoid the overfitting risk. As our DSR corpus is imbalanced, we applied random downsampling to control the distribution between the relation classifications. In previous studies, this approach achieved the highest performance at all levels of imbalance [54]. In addition, because differences in entity order can affect the performance of the relation classification model [55,56], we designated the order of drug and suicide entities in the relation class. For example, if the drug entity (e1) preceded the suicidal entity (e2) in a sentence, the sentence was designated as “e1-e2”; otherwise, it was designated as “e2-e1.”

The performance of the relation classification model is also affected by the properties of the data set. Therefore, we constructed various data sets with different properties from our DSR corpus and compared the model performances on each data set.

Table 1 lists the properties of the data sets used in this study. The data set properties are the *split ratio for training and test data sets, categorization of relation classifications*, and *order of entity mentions (within a sentence)*.

**Table 1:** Eight data sets constructed from our drug-suicide relation (DSR) corpus and their respective properties.

Data set	Split ratio for training and test data sets (training:test)	Categorization of relation classifications	Order of entities
1	90%:10%	None and ADE^a^	No
2	80%:20%	None and ADE	Yes
3	90%:10%	None and ADE	No
4	80%:20%	None and ADE	Yes
5	90%:10%	None, ADE, suicide means, and treatment	No
6	80%:20%	None, ADE, suicide means, and treatment	Yes
7	90%:10%	None, ADE, suicide means, and treatment	No
8	80%:20%	None, ADE, suicide means, and treatment	Yes

^a^ADE: adverse drug event.

#### R-BERT Model and Evaluation Metrics of Relation Classification

A suicide-drug relation class in a sentence containing an entity pair was predicted using the relation classification model R-BERT [46]. The R-BERT model enriches the pretrained BERT [45] model with entity information for relation classification by placing a special token at the beginning and end of each entity. In this study, vanilla BERT [45], BioBERT [57], PubMedBERT [58], ClinicalBERT [59], and SciBERT [60] LMs were used as the embedding layers of R-BERT. We fine-tuned the resulting R-BERT variations in 10 epochs and increased the maximum sentence length to 512, which is a limitation of the BERT model [45]. A 10-fold cross-validation of all data sets was performed using the *Stratified Shuffle Split* method provided in the *sklearn* library (version 1.0.2; [61]).

The performances of the relation classification models on ADE classes were evaluated in terms of the *F*_1_-score, defined as the weighted average of precision (ratio of correctly predicted positive observations to all predicted positive observations) and recall (ratio of correctly predicted positive observations to all observations in the actual class). The *F*_1_-score is considered as the gold standard of relation extraction, relation classification, and other NLP tasks. In the present evaluation, the true class was the ADE class and the false class was the non-ADE class.

## Results

On the basis of the titles and abstracts of 17,017 articles collected from PubMed, we created a corpus of 11,894 sentences with drug-suicide entity pairs and their relation classes.

Table 2 presents the frequencies of sentences in each relation class of the DSR corpus. The most frequent relation classes are “miscellaneous” (4250/11,894, 35.73%) and “none” (3761/11,894, 31.62%). The most common relation class is “Suicide means” (1726/11,894, 14.51%) followed by “treatment” (1281/11,894, 10.77%) and “ADE” (876/11,894, 7.36%). In the sentences of the “none,” “ADE,” and “treatment” classes, the “e1-e2” order appears more frequently than the “e2-e1” order. In the sentences classified as “suicide means” and “miscellaneous,” the order was similarly distributed between “e1-e2” and “e2-e1.”

Table 3 presents the top 10 most frequently mentioned drugs and their respective relation classes in the sentences of our DSR corpus (listed are the numbers of drug names, not the numbers of sentences). The most frequently mentioned ADE drug was isotretinoin (34/717, 4.7%), followed by varenicline (33/717, 4.6%), fluoxetine (30/717, 4.2%), and paroxetine (29/717, 4%). In the “suicidal means” category, the most commonly mentioned drug is insulin (63/1549, 4.07%). In the “treatment relation class,” the most commonly mentioned drugs are lithium (331/1042, 31.77%) and ketamine (261/1042, 25.05%). Most of the “treatment” drugs were among the top 10 drugs in “ADE.” Next, we explored the embedding LM that best improved the relation classification performance of the R-BERT model fine-tuned with our corpus.

Table 4 shows the performances of various R-BERT models with different embedded LMs after refinement on distinct data sets (Table 1 describes the properties of the data sets derived from our corpus). The *F*_1_-score of the R-BERT models ranged from 0.8781 to 0.9583. Overall, BioBERT predicted the ADE class better than the other embedding models, with an average *F*_1_-score of 0.9362. BioBERT also achieved the highest *F*_1_-score across 6 of the 8 data sets (the exceptions were data sets 5 and 8). Even in the exception cases, BioBERT achieved near-optimal performance. BioBERT was closely lagged by PubMedBERT (average *F*_1_-score=0.9238), which did not perform optimally across all the individual experiments.

Among the different data sets, data set 1 achieved the highest average *F*_1_-score. Data set 1 ignores the entity order and uses a 90% split ratio and a binary class (0.9498; see the *Average* column in Table 4). Meanwhile, 4 out of the 5 LMs achieved their highest *F*_1_-score when fine-tuned on data set 1 (BioBERT, 0.9583; PubMedBERT, 0.9503; ClinicalBERT, 0.9519; and SciBERT, 0.9496).

**Table 2:** Frequency of sentences (N=11,894) in each relation class in our drug-suicide relation (DSR) corpus (“suicide means” is the most common relation class).

Class and ordered entity pair of drug and suicidal entity			ID		Value, n
**No relation (n=3761, 31.62%)**			0		
	No relation (e1-e2)			2226	
	No relation (e2-e1)			1535	
**ADE^a^ (n=876, 7.37%)**			1		
	DRUG-ADE (e1-e2)			512	
	DRUG-ADE (e2-e1)			364	
**Means (n=1726, 14.51%)**			2		
	Means-event (e1-e2)			844	
	Means-event (e2-e1)			882	
**Treatment (n=1281, 10.77%)**			3		
	Treatment-event (e1-e2)			890	
	Treatment-event (e2-e1)			391	
**Miscellaneous (n=4250, 35.73%)**			9		
	Miscellaneous (e1-e2)			2141	
	Miscellaneous (e2-e1)			2109	

^a^ADE: adverse drug event.

**Table 3:** Top 10 drugs in each relation class of our drug-suicide relation (DSR) corpus (m: # of sentences mentioning an associated drug name).

Rank	Total (*m*=3308)		ADE^a^ (*m*=717)		Means (*m*=1549)		Treatment (*m*=1042)	
	Drug	Count	Drug	Count	Drug	Count	Drug	Count
1	Lithium	354	Isotretinoin	34	Insulin	63	Lithium	331
2	Ketamine	264	Varenicline	33	Paracetamol	56	Ketamine	261
3	Clozapine	168	Fluoxetine	30	Barbiturates	38	Clozapine	141
4	Insulin	67	Paroxetine	29	Metformin	38	Fluoxetine	22
5	Fluoxetine	58	Cocaine	25	Caffeine	25	Buprenorphine	21
6	Paracetamol	57	Zolpidem	22	Colchicine	25	Esketamine	16
7	Barbiturates	43	Rimonabant	17	Amitriptyline	19	Paroxetine	9
8	Metformin	40	Venlafaxine	15	Analgesics	19	Olanzapine	9
9	Cocaine	39	Lithium	14	Diazepam	19	Milnacipran	7
10	Paroxetine	39	Clozapine	13	Nicotine	16	Antidepressants	6

^a^ADE: adverse drug event.

**Table 4:** Performance comparison of various R-BERT (Bidirectional Encoder Representations from Transformers) models built by (1) applying different language models (LMs) as embedding layers and (2) fine-tuning different data sets.

Data set	Vanilla BERT [45]	BioBERT [57]	PubMedBERT [58]	ClinicalBERT [59]	SciBERT [60]	Average
1	0.9389	0.9583	0.9503	0.9519	0.9496	0.9498
2	0.9435	0.9528	0.9487	0.9489	0.9459	0.9480
3	0.9360	0.9522	0.9447	0.9432	0.9497	0.9451
4	0.9406	0.9486	0.9372	0.9448	0.9468	0.9436
5	0.9093	0.9223	0.9039	0.9262	0.9179	0.9159
6	0.8847	0.9268	0.9143	0.9102	0.9113	0.9095
7	0.8965	0.9194	0.9059	0.9132	0.8961	0.9062
8	0.8781	0.9089	0.8856	0.9162	0.9100	0.8998
Average	0.9159	0.9362	0.9238	0.9318	0.9284	N/A^a^

^a^N/A: not applicable.

Table 5 presents the performance results of the R-BERT models in terms of the different properties of the 8 data sets. The average *F*_1_-score for each property was determined from all the individual experimental results. When the training:testing split ratio of the data set was 90%:10%, the average *F*_1_-score was 0.9297, which was only 0.49% higher than that of the 80%:20% split ratio (*F*_1_=0.9248). This performance difference is minor. On average, the models performed 3.88% better in the binary class (*F*_1_=0.9466) than in the quaternary class (*F*_1_=0.9078). This result indicates a need to improve the performance of *n*-ary classification when *n* is >2. Finally, learning the order of the entities (0.9260) improved the performance by 0.24% compared with ignoring the ordering (0.9260), which is consistent with earlier findings [55,56]. The same tendencies frequently appeared in the precision and recall results (Multimedia Appendix 4).

**Table 5:** Average performances of the R-BERT (Bidirectional Encoder Representations from Transformers) models on data sets with different properties (the binary relation data set yields the best *F*_1_-score).

Data set properties and category		*F*_1_-score, mean (SD)
**Split ratio for training and test data sets**		
	90%:10%	0.9297 (0.0078)
	80%:20%	0.9248 (0.0078)
**Relation set**		
	Binary relation set	0.9466 (0.0048)
	Quaternary relation set	0.9078 (0.0110)
**Ordered entity pair of drug and suicidal entities**		
	Yes	0.9284 (0.0058)
	No	0.9260 (0.0103)

## Discussion

### Principal Findings

To our knowledge, this is the first and largest data set of DSRs. The existing data sets include information on ADEs but do not focus on drug-suicide ADEs; thus, they deliver insufficient data on drug-suicide associations. Among the 6821 sentences on drug-related adverse events in the MEDLINE corpus, only 3 (0.04%) contained an entity related to suicide. In contrast, our corpus contained a large number (876) of entities uniquely relating suicide as an ADE.

A valuable data set must contain sufficient data. When collecting the titles and abstracts containing information on DSRs, we applied a detailed search query using both MeSH and text words. The MeSH term was particularly useful when searching for a wide range of articles in PubMed. Previous studies used only MeSH terms when searching PubMed for corpora. However, the indexing time of MeSH is likely to miss the latest relevant articles [62]. DeMars and Perrusso [63] compared the precision and recall of each strategy after searching for relevant articles using MeSH and text words in PubMed. They recommended combining MeSH and text words to obtain the most comprehensive number of papers.

Manual annotation is time-consuming, costly, and laborious. Although MeSH and text words garnered the titles and abstracts from articles mentioning drugs and suicidal behaviors, it could not guarantee that every sentence was suicide related. To address this problem, we filtered the sentences classified as suicide relevant using a pretrained zero-shot classifier. In other words, we checked whether the classifier assessed the given sentences as suicide related and contained suicidal keywords. Consequently, only 6.9% (11,894/172,249) sentences collected from PubMed included relevant information for the DSR corpus. This new approach effectively reduced the data that could be annotated and provided a new strategy for preannotations. To reduce the annotation effort, previous studies randomly sampled the initial documents [15,18,20-22], restricted the publication date of the documents [18,22], or filtered the initial documents based on some required properties [18]. These techniques risk decreasing the quantity of fundamental data that can be collected and annotated.

Some of the top 10 drugs associated with ADEs (fluoxetine, paroxetine, venlafaxine, lithium, and clozapine) were also classified as treatment drugs. This tendency may reflect the ongoing controversy on the association between suicide and drugs administered to patients with mental health disorders. Some representative studies have reported that SSRIs effectively prevent suicidal risk, whereas others have reported that such drugs potentially increase the suicidal risk [64]. Furthermore, medication adherence is an important determining factor for successful pharmacotherapy for mental disorders. To fill this data gap, diverse methods for real-time monitoring of medication adherence using the medical devices have been recently reported [65].

We also evaluated the performance of the R-BERT relation classification model with several pretrained LMs as the embedding layers. After pretraining on PubMed, R-BERT provided a slightly higher relation classification performance on the corpus with BioBERT than with PubmedBERT. This tendency can be explained either by the larger pretraining vocabulary of BioBERT than that of PubmedBERT or the continuous pretraining process of BioBert from the base LM [58] (whereas PubmedBERT was pretrained from scratch). Increasing the pretraining data set and vocabulary increases the diversity of the patterns that a model can learn. The results indicate that BioBERT maintains the base vocabulary during ongoing pretraining and uses the base (Vanilla BERT) weights as the initial weights.

Concerning the data set properties, the performance was maximized when the data set was split into a 90%:10% training:testing ratio, when the classification scheme was binary, and when the entities were ordered. More importantly, all tested models classified the drug-suicide relationships with *F*_1_-score around 0.9 after fine-tuning on our corpus, higher than on the available corpora. For example, Gurulingappa et al [21], who dealt with sentence classification, reported an *F*_1_-score of 0.70 after training MaxEnt on the MEDLINE corpus. Kim et al [66], who dealt with key sentence extraction, trained the BERT classification model on the Drug-Food Interaction corpus of drug and food interactions, obtaining *F*_1_-score from 0.506 to 0.738. The varied scopes and sizes of corpora and the different types of classification models preclude a direct comparison of results of this study with those of the previous studies. Nevertheless, this result clearly demonstrates the value of our corpus in NLP tasks.

These results were obtained through experiments on a specific type of ADE but appear to be applicable to other drug-related adverse events. All nondrug entities were linked to suicide in our research, but the portion of the corpus having the assigned ADE relation can (in theory) be used to investigate drug adverse events not related to suicide. In practice, applying a specific type of ADE to a broader ADE task may decrease the overall performance or change the performances of different LMs. Masking the events in BERT_MTB+EM_ [47] might reduce the effect of suicide-related bias, but eliminating the bias through event masking is difficult because specific words cueing the suicidal nature of an entity may remain in the context; for example, a sentence with the entities excluded can retain the term “attempted.”

This corpus is extendible to the development of other NLP systems. For instance, an automatic extraction system accessing our corpus can obtain additional information on the drug-suicide association, such as treatment of suicidal ideation and drugs used in suicide attempts. Our DSR corpus contains sufficient data on the DSR not only for “ADEs” but also for “suicidal treatment” and “means” (14.5% and 10.8% of the corpus, respectively). Moreover, the newly discovered suicide-related entity can complement the existing named entity recognition tools.

### Limitations

There are some drawbacks to this study. First, the ADEs are more narrowly distributed than other relation categories, leading to potential class imbalances when developing relation classification systems using the corpus. To alleviate these problems, we performed downsampling [67] and eliminated the sentence duplicates before applying the relation classification model to various data sets generated from our corpus. We expect this treatment to offset the negative effects of the class imbalance. Solving for the class imbalance issues is beyond the scope of this work but should be addressed in future work. For the same reasons, we did not explore the noisy miscellaneous class, which reveals little information on DSRs. The “Miscellaneous” class is also worthy of investigation in future studies. Note that this work concentrated on building the data set and assessing its suitability in performance evaluations. Moreover, we restricted the sentence length to 512 characters (the upper limit of BERT encoding), but this restriction could be relaxed for NLP jobs that do not use BERT. This study excluded overlaps between drugs and suicidal events. Finally, because this corpus was created solely from academic literature, its scope may not extend to social media.

### Conclusions

Extracted from research articles, this developed DSR corpus is the largest and most comprehensive corpus for drug-suicide entities and their relations (Multimedia Appendix 5). After confirming the consistency of the annotations in the DSR corpus, we applied a new approach for reducing the load of manual annotations. When fine-tuned on our corpus, all R-BERT models achieved competitive performance with *F*_1_-score above or only slightly below 0.9. We believe that our corpus can be widely used for developing automatic information extraction systems and for activating relevant research on DSRs.

In future, we plan to expand the data set by revising ambiguous cases and diversifying the ADE class into 6 subclasses [68]. We will also cover colloquial text sources from Twitter and other social media sites.

## Appendix

Multimedia Appendix 1 Sentences with suicidal entities from the MEDLINE adverse drug event data set.

Multimedia Appendix 2 Comparison with the previous corpus with adverse drug events (ADEs) annotations.

Multimedia Appendix 3 Annotation guidelines of the drug-suicide relation corpus.

Multimedia Appendix 4 *F*_1_ score, precision, and recall results of the R-BERT (Bidirectional Encoder Representations from Transformer) model differentiated by embedding-layer language models and fine-tuning data sets.

Multimedia Appendix 5 Drug-suicide relation corpus.

## Abbreviations

ADE: adverse drug event
BART: Bidirectional and Auto-Regressive Transformers
BERT: Bidirectional Encoder Representations from Transformers
DSR: drug-suicide relation
LM: language model
MeSH: Medical Subject Headings
NLP: natural language processing
SSRI: selective serotonin reuptake inhibitor
