# Supplementary material for: Supervised Relation Extraction Between Suicide-Related Entities and Drugs: Development and Usability Study of an Annotated PubMed Corpus
Source: J Med Internet Res. 2023 Mar 8;25:e41100. doi: 10.2196/41100 (PMC10034613; doi:10.2196/41100)
Supplement: Multimedia Appendix 2 [file jmir_v25i1e41100_app2.docx]

Table 1. Comparison with the previous corpus with ADEs annotations.

| **Title** | **Type of document** | **Data source** | **Annotated entities** | **Annotated relations** | **Size**  **(number of sentences /**  **number of sentences with suicide-related ADEs)** |
| --- | --- | --- | --- | --- | --- |
| ADRMine dataset [23] | Sentences from Tweets and Posts | Twitter, DailyStrength | signs/  symptoms (including adverse drug reaction) | none | 1784 / Don’t know |
| EU-ADR [15] | sentences from title and abstract | MEDLINE | drug, disease | drug-disease (incl. ADE) | 668 / Don’t know |
| MEDLINE ADE [21] | sentences from abstract | MEDLINE | drug, condition (e.g. diseases, signs, symptoms) | drug-ADE | 6821 /  3 |
| TwiMed [22] | Tweets, PubMed sentences | Twitter, PubMed | incl. drug, symptom, disease, status, sentiment | incl. outcome-negative | 2000 /  Don’t know |
| **Our work**  **(DSR)** | **Titles and abstracts of biomedical articles** | **PubMed** | **Drug, suicide** | **Drug-suicide relations** | **11894/**  **876** |
