# Supplementary material for: Supervised Relation Extraction Between Suicide-Related Entities and Drugs: Development and Usability Study of an Annotated PubMed Corpus
Source: J Med Internet Res. 2023 Mar 8;25:e41100. doi: 10.2196/41100 (PMC10034613; doi:10.2196/41100)
Supplement: Multimedia Appendix 3 [file jmir_v25i1e41100_app3.pdf]

경북대학교
KYUNGPOOK NATIONAL UNIVERSITY

DSR PubMed 1.0 Annotation guidelines

version from 2022-07-05

Introduction

DSR (Drug-Suicide Relations) PubMed corpus is an annotated corpus of drug entities (DE), suicide-related entities (SE), and relation classes between them. The corpus consists of titles and sentences from PubMed abstracts.

The corpus consists of single sentences and supports only inter-sentential relations. All the evidence should appear in the same sentence.

For more information about the corpus constituents and the algorithm of the data collection, please refer to the original paper.

Entities

An entity can be a single word or phrase that appears in the annotating text. In this guideline, we focused on two types of entities: drugs and suicidal entities. This document provides the guidelines followed during the annotating process of drug entities, suicidal entity, and their relations.

Drug

Drug Entities are pre-annotated in the text by means of an automatic NER model (Med7[1]). The model recognized enzymes, amino acids, hormones, raticides, veterinary drugs, herbicides, and pesticides as a drug names as well. Next are the corrections made by annotators after NER.

Corrections after NER

1. Some drugs were additionally annotated. For example, narcotic drugs (marijuana, heroin, etc.), acetaminophen, drug classes (SSRIs, TCAs, antidepressants, analgesics, etc.), and pharmaceutical product names (Coricidin, Prothiaden, etc.).

Sentence	Med7	DE
Findings suggest that differences exist based on demographic variables (gender, age, race, and sexual orientation), lifetime drug use (inhalants, Valium™, crack cocaine, alcohol, Coricidin™, and morphine), recent drug use (alcohol, ecstasy, heroin, and methamphetamine), mental health variables (suicide attempts, familial history of substance use, and having been in substance abuse treatment), and health variables (sharing needles and having been tested for HIV).		Valium™

2. A missing part of entities was filled in.

Sentence	Med7	DE
Massive ingestion of isosorbide-5-mononitrate and nitroglycerin: suicide attempt by an adolescent girl without previous heart disease.	methamphetamine	isosorbide-5-mononitrate

3. The parenthesized additional information (such as brand or abbreviation), was included in a label.

Sentence	Med7	DE
Methamphetamine (MAP) abusers are commonly noted as having psychosis, depression and suicidal behavior.	isosorbide	Methamphetamine (MAP)
Several reports were published in the psychiatric literature in 1990 and 1991 documenting fluoxetine (Prozac) causing patients to consider or attempt suicide.	fluoxetine	fluoxetine (Prozac)

4. In case of overlapping recognized DEs, keep only the broader one.

Sentence	Med7	DE
Deaths including those from natural causes, toxicity, accident and suicides with positive forensic toxicology analyses for methamphetamine and its metabolite amphetamine in postmortem samples were investigated.	amphetamine	
	methamphetamine	methamphetamine

5. If a drug and its metabolites are recognized together, the drug is excluded.

Sentence	Med7	DE
Deaths including those from natural causes, toxicity, accident and suicides with positive forensic toxicology analyses for methamphetamine and its metabolite amphetamine in postmortem samples were investigated.	amphetamine	
	methamphetamine	methamphetamine

Suicidal Entity

For suicidal entities, the mention of suicide-related events, tendencies, and behaviors, including suicide risk, suicidal attempt, completed suicide, and suicidal ideation or behavior disorders were annotated. The entities are nominals (nouns or noun phrases), related to suicide. Next are elaborations on controversial cases.

1. "Risk" etc expressions are not included in a label if appears before the suicide-related word or phrase.

Sentence	SE
To compare the risk of suicide in adults using the antidepressant venlafaxine compared with citalopram, fluoxetine, and dothiepin.	suicide
In treatment responsive BD patients, lithium (Li) stabilizes mood and reduces suicide risk.	suicide risk

2. In case of concatenation by "/" , "or" , "and", separate labels only if the meaning will remain the same after separation. If the meaning changes after separations, the labels are not separated and the conjunctions are included in the label.

Sentence	SE
The use of paroxetine may increase suicidal behavior and suicidal ideation.	suicidal ideation
	suicidal behavior
Here we add further Associations of Z-drugs, trazodone, and sedative benzodiazepines (temazepam, triazolam, flurazepam) with suicidal ideation, planning, and attempts were estimated using binomial logistic regression.	suicidal ideation, planning, and attempts

3. Negated entities were annotated as well.

Sentence	SE
Moreover, convincing evidence exists that lithium has added value and benefit for its unique anti-suicidal effects as well as reducing mortality by other causes.	anti-suicidal effects

4. Abbreviations are annotated as well. When an abbreviation appears next to the full expression, the abbreviation is annotated together with the full expression.

Sentence	SE
After adjusting for demographic characteristics, tobacco use, family history of suicide and depression, both SI and SA were positively associated with AUD, cannabis and cocaine use.	SI
	SA
Suicidal ideation (SI) is often cited as a reason to exclude patients from interferon-based treatment or to terminate antiviral treatment that is in progress.	Suicidal ideation (SI)

5. Expressions that do not contain a stemmed version of "suicide" ('suicid'), but suggest suicidal action in form of self-harm, self-poisoning, or fatal intent.

Sentence	SE
Although anecdotal, our case report points toward safety of pregabalin following deliberate self-poisoning.	self-poisoning

Relations

Relation represents existing one-to-one connection between drug and SE. Annotated types of relations are: 'adverse drug events (ADE)', 'suicide means', 'treatment', 'miscellaneous', and 'none'. The annotation does not depend on the origin-entity and the end-entity order. List of relations and their definitions is provided in the table below

ID	Relation class	Description
0		The relation class designates that there is no relation between entities.<br>1. The SE is not related to suicide (suicide vector, cell suicide, etc.)<br>2. The DE is not mentioned as a drug in the sentence, but indicator, biomarker, etc.
1	Adverse drug event	The relation class designates the occurrence of suicidal events after drug administration, regardless of how clear the relation is.<br>1. Explicit relation of DE with suicidal behavior.<br>2. Increased suicidal risk after DE administration.<br>3. Suicidal behavior as a withdrawal effect of DE.<br>4. Suicidal behavior induced by DE.
2	Suicide means	The relation class designates the deliberate use of drugs (overdose, etc.) for suicidal purposes.<br>1. Explicit DE administration for suicidal purposes.<br>2. Implicit DE administration with suicidal purposes.<br>2.1. The sentence contains keywords, designating DE administration for suicidal purposes (suicidal ingestion, suicidal overdose, suicidal exposure, self-poisoning, etc.)<br>2.2. The DE described using “suicidal agent”-like keywords.
3	Treatment	The relation class designates that the DE is used to treat signs or symptoms of suicidal ideation and/or suicidal behavior.<br>1. The DE is prescribed to a person with high suicidal risk.<br>2. The DE with mentioned anti-suicidal effect.<br>3. Decreased suicidal thoughts after DE administration.<br>4. Sentence mentioning a possibility of suicidal behavior decrease.
9	Miscellaneous	The relation class designates that the relation between entities is not suitable to any of the classes above.<br>1. Comparative sentences (comparison of therapeutic effects or side effects between drugs, placebo-controlled experiments, comparison between patient groups, etc.).<br>2. Purpose of a study (article’s title, study description, etc.).<br>3. Explicit relation absence between DE and SE.<br>4. Sentence where the relation’s existence is uncertain.

References

1. Kormilitzin A, Vaci N, Liu Q, Nevado-Holgado AJ. Med7: a transferable clinical natural language processing model for electronic health records. Artificial intelligence in medicine. 2021;118:102086.
